# How to clone a Dire Wolf?

**DOI:** 10.1038/s44319-025-00474-w

**Published:** 2025-05-16

**Authors:** Jacob Höglund

**Affiliations:** Department of Ecology and Genetics, Uppsala University, Uppsala, Sweden

**Keywords:** Biotechnology & Synthetic Biology, Evolution & Ecology

## Abstract

The recent reports about the ‘resurrection’ of the dire wolf - an animal that went extinct more than 10 000 years ago, has been hailed as a landmark in resurrecting extinct species. Despite the impressive science behind it, it is not a dire wolf and it is not a solution to the ongoing biodiversity crisis.

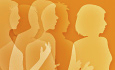

The company Colossal Biosciences reported in April that they had resurrected or, if you so wish, de-extincted, an extinct animal, the Dire wolf—to be more specific *Aenocyon dirus*. *Aenocyon* disappeared about 10,000 years ago along with most of its prey species, Late Pleistocene megaherbivores. By sequencing DNA from fossil finds of this species (Gedman et al, 2025, preprint), Colossal went on and used CRISPR/Cas9 genome editing technology, to transplant 14 DNA sequences from the sequenced Dire wolf genome into embryos of their closest living relative, the Grey wolf. Subsequently, three large Grey wolfs with pale pelages were born by surrogate mothers whom were domestic dogs. Admittedly, this is an impressive achievement showing how gene editing can be used to transform the phenotypes of complex organisms that may make look like something that used to walk the Earth in the past.

But is this really de-extinction? Have Colossal made use of the latest scientific technologies to help solve one of the biggest problems facing modern society: the biodiversity crisis? Since life formed on Earth about 3.6 billion years ago, biodiversity has been increasing. Many, in fact most, have gone extinct but throughout the history of life on Earth more and more species have inhabited the planet. There are six major exceptions though. The Earth has faced five mass extinctions in the past, during which biodiversity declined. One of the most well-known happened at the end of the Cretaceous period about 65 million years ago, when the dinosaurs—except the descendants of modern birds—disappeared. These previous mass extinctions have a scientific explanation. Geologists have identified major geophysical events, such as impacts by large meteorites or massive, long-term volcanic eruptions, which had major climatic effects, causing the mass extinctions. At present, we see similar trends whereby net biodiversity is being lost rather than accumulating (Waters et al, 2016). The exception is that this mass extinction event is caused by human action. Anthropogenic actions such as over-exploitation, habitat destruction, climate change, and pollution are causing the present-day species loss. Thus, government and non-government agencies throughout the globe try to prevent further biodiversity loss and to preserve species that are facing extinction. The traditional way to do this is to identify threatened species, preserve healthy populations, and protect their habitats.

Why am I mentioning this when writing an opinion piece on the Dire wolf? Well, it has been claimed, though admittedly not by Colossal and other commercial companies involved in similar activities, at least not when you press their scientists on the issue, that we could resurrect extinct species by bioengineering. However, the perception by the public and even government officials is that, no matter human actions, we can simply resurrect extinct species if it is lost. Even if Colossal admits that this is a naïve interpretation, they state that the creatures they aim at creating would fill the ecological niches once used by the extinct species and that their efforts would help to solve the biodiversity crisis. This is absolutely wrong in my mind. There is instead a risk that less money is allocated for biodiversity protection and it gives a false impression that there is no need to preserve habitats.

Let us be clear on one thing: what’s gone is gone and cannot be brought back. When the last individual of a unique genetic lineage dies, with it goes a complex interplay of genes shaped by their evolutionary history whereby both chance events and adaptations to their environment played important parts. What we know from modern conservation science is that for species to be able to cope with future challenges, their population sizes need to be considerable. Large populations harbor a greater suite of genetic variants which make it more likely to be able to cope with present and future challenges, more so than small populations that have lower genetic diversity. Species with large populations with lots of genetic variation are hence more resilient.

By creating three individuals that look like Dire wolves, Colossal have made just that. Dire wolves and Grey wolves are genetically very different species. Colossal’s own study showed at least 15 million genetic variants—this is a conservative estimate as the genomes are not complete—between the two species. In all, 14 loci is quite a long way from 15 million variants. Therefore, the company have not resurrected the Dire wolf. Furthermore, these three Grey wolves with Dire wolf genes pasted into their genomes are very far from what could be considered a healthy free-living population. Let us not forget that the reason why the Dire wolves disappeared was the disappearance of megaherbivores on which they preyed. The Dire wolf lineage is gone and even if we could multiply the newborn pups—which in reality are genetically modified Grey wolves—how would we maintain a free-living population? How would we prevent them from back-crossing with Grey wolves or even dogs which in genetical terms are also wolves? Would they have enough genetic variation to be able to cope with future challenges? I think not.

Finally, an explanation of the title of this piece is called for. One front-figure of Colossal, Dr. Beth Shapiro, wrote a book in 2016 called “How to clone a Mammoth”, which I very much enjoyed reading. Therein, she explains the arguments put forth here. What is gone is gone. It is impossible to clone a mammoth for various technical reasons explained in the book even if we can read their genetic code by using ancient-DNA technology. What can be done is to gene-edit a contemporary genome into something that may look like a mammoth, or a dire wolf or whatever we wish to do but it will at best be a hairy Asian elephant or a big pale Grey wolf. Not a “de-extincted” species. Therefore, instead of wasting efforts and resources on vanity projects, it is better to protect existing biodiversity; bioengineering will not solve the biodiversity crisis.

## Supplementary information

Peer Review File
