# Supplementary material for: How to clone a Dire Wolf?
Source: EMBO Rep. 2025 May 16;26(12):2969–70. doi: 10.1038/s44319-025-00474-w (PMC12187906; doi:10.1038/s44319-025-00474-w)
Supplement: Supplementary file 1 — Peer Review File [file 44319_2025_474_MOESM1_ESM.pdf]

EMBO reports

How to clone a Dire Wolf?

Jacob Höglund

Corresponding author(s): Jacob Höglund (jacob.hoglund@ebc.uu.se)

Review Timeline:

Submission Date:	5th May 25
Accepted:	5th May 25

Editor: Holger Breithaupt

Transaction Report: This article type is not peer reviewed.
